# Enhancement of the Performance and Data Processing Rate of an Optical Frequency Domain Reflectometer Distributed Sensing System Using A Limited Swept Wavelength Range

**DOI:** 10.3390/s18103480

**Published:** 2018-10-16

**Authors:** Kunpeng Feng, Jiwen Cui, Yihua Jin, Xun Sun, Dong Jiang, Hong Dang, Yizhao Niu, Jiubin Tan

**Affiliations:** 1 Institute of Ultra-precision Optoelectronic Instrument Engineering, Science Park, Harbin Institute of Technology, Harbin 150080, China; koiefh@163.com (Y.J.); sx2503962673@163.com (X.S.); bblong93@163.com (D.J.); sired_hit@163.com (H.D.); m18846819526@163.com (Y.N.); jbtan@hit.edu.cn (J.T.); 2 Department of Automation, Harbin Engineering University, Harbin 150001, China; 3 Huawei Technologies Limited Company, Huawei Building, Beijing 100085, China

**Keywords:** distributed sensing, Rayleigh scattering, optical fiber, least-squares method

## Abstract

A novel optical frequency domain reflectometer (OFDR) processing algorithm is proposed to enhance the measurable range and data processing rate using a narrow swept spectrum range and reducing the time consuming of the process distributed sensing results. To reduce the swept wavelength range and simultaneously enhance strain measurable range, the local similarity characteristics of Rayleigh scattering fingerprint spectrum is discovered and a new similarity evaluation function based on least-square method is built to improve the data processing rate and sensing performance. By this method, the strain measurable range is raised to 3000 µε under a highest spatial resolution of 3 mm when the swept spectrum range is only 10 nm and the data processing rate is improved by at least 10 times. Experimental results indicate that a nonlinearity of less than 0.5%, a strain resolution of better than 10 µε, a repeatability at zero strain of below ±0.4 GHz and a full-scale accuracy is lower than 0.85 GHz under a highest spatial resolution of 3 mm can be achieved. Advantages of this method are fast processing rate, large strain measurable range, high SNR, and applicability with current OFDR systems.

## 1. Introduction

In the areas of non-destructive measurement and structural health monitor, optical frequency domain reflectometers (OFDRs) have been widely utilized for many years because of their excellent spatial resolution and high strain accuracy [1,2]. Over these years, OFDRs based on the Rayleigh scattering (RS) spectrum gave gradually received increasing attention with the progress of the investigation of Rayleigh backscatter spectra in optical fibers. Rayleigh backscatter in optical fibers is induced by the fluctuations of the index of refraction profile along the fiber. The RS spectrum, just like a “fingerprint”, is stable and unique once the fiber is manufactured [3]. Also, a relation between the measurable strain of the fiber gauge and the inerratic fingerprint spectrum shift has been established for distributed strain sensing [4]. The spectrum shift is usually achieved by combining OFDR with cross-correlation of the RS fingerprint spectra. Thus, even a common single mode fiber (SMF) without FBGs can be employed as a distributed sensor [3,5].

However, in practice, the sensing accuracy of distributed strain, even the feasibility of this method is based on the similarity between RS reference spectrum (ReS, with known distributed strain) and RS measurement spectrum (MeS, with unknown distributed strain). Furthermore, the swept spectrum range should be expanded to enhance the measurable strain range. The data processing rate is thus limited by the sweep time and large amount of data. Otherwise, the similarity will significantly degenerate when the loaded strain is over 1000 µε and swept wavelength range is 10 nm once the spatial resolution is downwards to several mm and spectrum resolution is downwards to several pm. With the degeneration of the similarity, fake peaks or multi-peaks start to appear in the cross-correlation results, which would yield mistakes in calculating spectrum shifts. OFDR based on RS fingerprint spectrum which can measure a distributed strain larger than 1000 µε and the swept range is less than 20 nm was rarely reported, especially under a high spatial resolution downwards to mm-level [6]. On the other hand, the Pearson correlation coefficient should be employed to evaluate the similarity between ReS and MeS. In this process, the conventional evaluation function is performed and it is very time-consuming when processing distributed sensing data of high spatial resolution and a long measurable range. The real-time performance needs to be improved.

In this paper, a novel OFDR processing algorithm is proposed to enhance the measurable strain range and data processing rate with a limited swept wavelength range of 10 nm. The strain is related to the spectrum shift. The motivation of this work is to solve the problem of invalidation of the conventional method when measuring a large strain and the failure to find the true cross-correlation peak when the sweep is insufficient. First, the degeneration mechanism of RS spectrum similarity is investigated and the local similarity characteristics of the RS fingerprint spectrum are discovered to improve the similarity between ReS and MeS under a large strain. Then, a new similarity evaluating function based on the least-squares method is built to replace the Pearson correlation coefficient and the calculation amount is significantly decreased. By combining these two variations, the proposed OFDR method matches the local ReS on the MeS to find the most similar MeS segment wherein the least-square method is employed to evaluate similarity. Therefore, a higher SNR during the determination of the spectrum shift and a faster data processing rate can be achieved. Experimental results indicate that SNR is improved from 1~2 to 2~12 and the data processing rate is 10 times faster than with the current method. The measurable strain range of the proposed method is improved up to 3000 µε (limited by maximum stretch of optical fiber) and the nonlinearity is less than 0.5% under a highest spatial resolution downwards to 3 mm. The strain resolution is better than 10 µε, the repeatability at zero strain is below ±0.4 GHz and the full-scale accuracy is less than 0.85 GHz under a highest spatial resolution of 3 mm, which is better than that of LUNA ODiSI-B representing the leading level of OFDR.

## 2. Sensing Principle

### 2.1. The Degeneration Mechanism of the RS Spectrum Similarity and the Local Similarity Characteristics

Monitoring the RS spectrum of the sensing fiber is commonly utilized to sense variations of the distributed strain along the sensing fiber in conventional OFDR methods. That is based on the characteristics of the sensing fiber that RS spectrum remains stable and unique once the fiber was manufactured and is only influenced by variations of the temperature or strain [7]. RS spectrum is induced by random fluctuation of the refractive index in the fiber, and its properties are similar to the fiber Bragg grating (FBG). Therefore, the RS can be considered as the weak random Bragg grating [3]. As the FBG’s spectrum varies with the loaded strain, the external strain also causes a shift of the RS spectrum. The ratio of spectrum shifts to strain variations is usually constant for a specific fiber. Thus, distributed strain sensing can be accomplished by monitoring the RS spectrum shift of the measured gauge.

**Figure 1:**
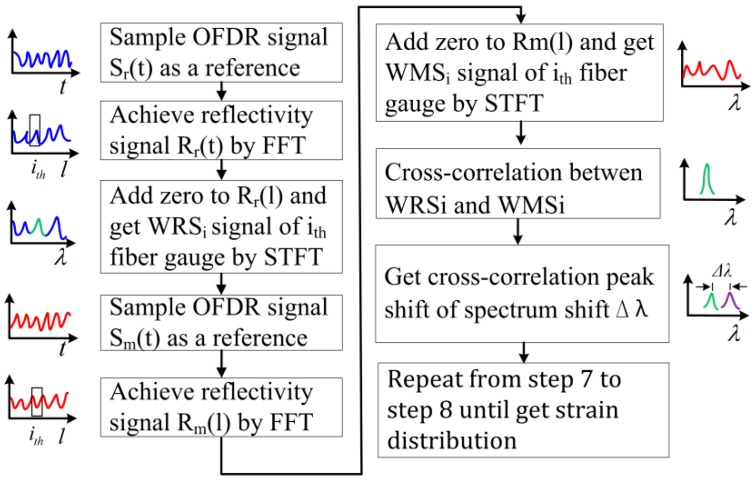
The flow diagram of conventional OFDR method. OFDR: optical frequency-domain reflectometry; Sr(*t*): the reference OFDR signal; Rr(*l*) the reference OFDR reflectivity signal; WRSi: whole reference spectrum of i-th fiber gauge; Sm(*t*): the measurement OFDR signal; Rm(*l*) the meaurement OFDR reflectivity signal; WMSi: whole measurement spectrum of i-th fiber gauge.

In the OFDR system, a tunable laser source working in linear-frequency-sweeping mode is utilized to demodulate the position information and distributed strain of every gauge. The beat signal consists of the RS light and local oscillator light. Based on properties of the linear frequency swept light source, the relationship between the position and the beat frequency is directly proportional. Therefore, position information can be achieved through transforming signals from time-domain into frequency-domain using fast Fourier transform (FFT). Accordingly, the RS spectrum located at different positions can be extracted by a short-time Fourier transform (STFT). The spatial resolution and the measured gauge position are controlled by setting the length and center position of the window of STFT. Then, the cross-correlation is used to calculate spectrum shift between the MeS (with unknown distributed strain) and the ReS (with known distributed strain). The achieved corresponding spectrum shift is proportional to the distributed strain. Theoretically, the distributed strain of the whole sensing fiber can be obtained by performing the STFT and the cross-correlation on every measured gauge along the fiber. The conventional OFDR method is illustrated in Figure 1. The cross-correlation between WRS_i_ and WMS_i_ can be expressed as:

(1) $$R(n)=\sum_{m=0}^{2N-1}{\mathrm{WRS}}_{i}(m)\cdot {\mathrm{WMS}}_{i}(m+n)$$

where, *n* = −*N*, −*N* + 1, …, *N* − 1, *N* is the length of the WRS_i_ and WMS_i_ sequences, and the elements WRS and WMS sequences are zeros when the index is not within [0, N].

The swept spectrum range should be expanded to enhance the strain measurable range which would induce a long sweep time and large amount of data. However, a broad swept spectrum range would limit the data processing and occupy a big memory when a high spectrum or strain/temperature resolution is required. When the swept spectrum range is *B*_1_ and the spectrum shift induced by suffered strain is *S*_1_, the different spectrum percentage between ReS and MeS is written as:

(2) $$p=\frac{B_{1}-S_{1}}{B_{1}}\times 100\%$$

The positive and negative shift of the reference spectrum is equivalent. So the positive is taken as an example to investigate the influence of the new segments. The reference spectrum is *R*(*n*) (wherein, *n* = 1, 2, 3, …, *K*) and the measurement spectrum *M_i_* can be expressed as:

(3) $$M_{i}=[N_{i}(m),R(n+m)]$$

where, *N_i_*(*n*) is the new spectrum segment, *n* = 1, 2, 3, …, *K* − *m*, *m* is the spectrum shift, and *m* ≤ 0.5*K*.

**Figure 2:**
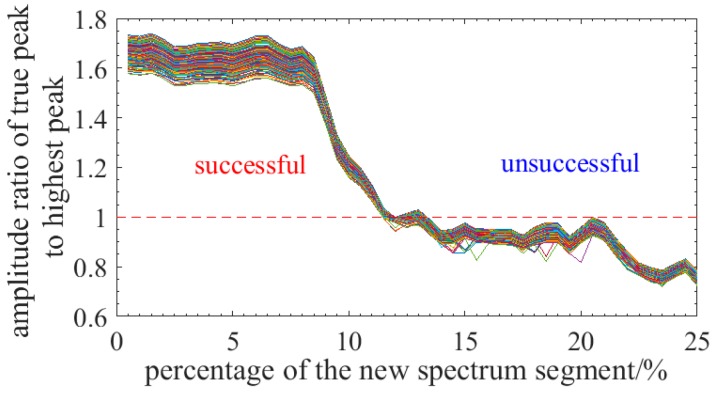
Statistical results of the influence of the parameter p on finding the true peak representing the spectrum shift.

Then, simulation is run to investigate the influence of the parameter *p* on finding the true peak representing the spectrum shift. In the simulation, experimental results of 180 reference spectra are statistically analyzed and new spectrum segments is randomly selected in the reference spectra (except the current reference spectrum). Figure 2 demonstrates the statistical results of the amplitude ratio of the peak to highest peak when the parameter *p* varies from 0 to 50% with a 10% step and the simulation is run 180 times under a same condition. Figure 2 demonstrates the results of 180 times and every curve represents an experiment. It can be concluded that the cross-correlation method is valid when the ratio is greater than 1 because the true peak is the highest and it can be determined by this method. Figure 2 indicates that the cross-correlation method is invalid when the different spectrum percentage between ReS and MeS is over ~13%. So the measurable strain is less than ~1100 με when the swept spectrum range is 10 nm.

**Figure 3:**
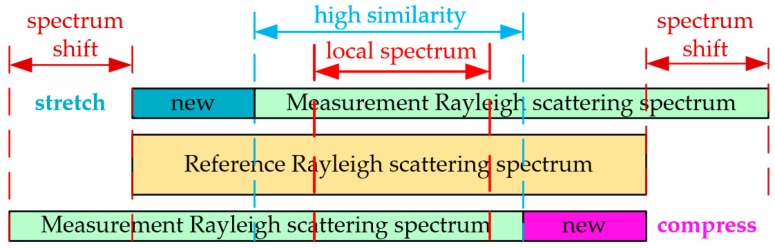
Schematic of the reason for the degeneration of similarity between MeS and ReS caused by the distributed strain.

This physical description is demonstrated in Figure 3 and it indicates that the MeS (0 με) has a high similarity with the ReS (0 με), however, the original MeS shifts relative to the ReS when the strain is loaded on the sensing fiber. New spectrum segment appears in the MeS spectrum and it has no corresponding similar spectrum segment in the ReS. The spectrum shift under a small strain load is relatively small, so that the proportion of new spectrum is not large enough to cause significant similarity degeneration. Therefore, it can be deduced that the low similarity between the MeS and the ReS is owing to the large spectrum shift induced by the large strain.

**Figure 4:**
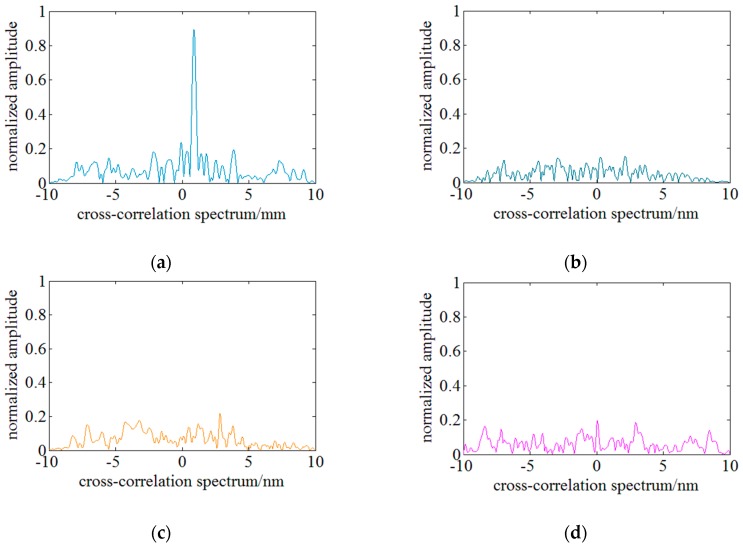
Cross-correlation results between ReS and MeS under different strain load. (**a**) the single peak under a strain of 700 µε. (**b**) multi-peaks under a strain of 1300 µε. (**c**) multi-peaks under a strain of 2100 µε. (**d**) the fake peak under a strain of 2300 µε.

The experimental data in Figure 4 are derived from distributed strain experiments in which the strain loads are 700 με, 1300 με, 2100 με and 2300 με when the swept spectrum range is 10 nm. In practice, however, this method only works well when the distributed strain is small. Experimental results indicate that SNR of the cross-correlation result degenerates significantly and multi or fake peaks begin to appear in the cross-correlation results when the loaded distributed strain exceeds 1000 µε.

In conventional OFDR methods, spectrum shifts are calculated by true peaks’ position in cross-correlation results. Under a small strain load, the true peak is usually unique and keeps highest amplitude. Figure 4a shows the ideal cross-correlation result and the true peak is unique and highest. Figure 4b,c shows that there are multiple peaks in the cross-correlation result and this will make the determination of the true peak difficult. Figure 4d shows that fake peaks refer to that there are some peaks which are higher than the true peak and will lead to wrong determination of the spectrum shifts. Based on the experimental data, peaks in cross-correlation results are relatively lower when multi or fake peaks begin to appear. As illustrated in Figure 3, the highest peak in the ideal cross-correlation results exceeds 0.8, while the highest peaks of multi and fake peaks are lower than 0.2. Therefore, it can be concluded that the appearance of multi and fake peaks is mainly caused by the degeneration of similarity between the MeS and ReS. With the increase of the loaded strain, the similarity between the MeS and ReS decreases significantly, and multi and fake peaks begin to appear in cross-correlation results. Finally, the determination of the spectrum shift is no more robust and becomes inaccurate. The strain measurable range of OFDR is thus limited.

During the experiments, the sweep wavelength range is 10 nm (~1200 GHz) and the spectrum shift caused by 3000 με is ~3.8nm. Thus, the new spectrum without similarity accounts for ~38% of the ReS under a strain of 3000 με, which will result in a significant decrease of similarity between the MeS and the ReS and the appearance of multiple or fake peaks. In summary, avoiding the appearance of multiple and fake peaks is mainly to rule out the influence of the new spectrum segment. On the other hand, there exists a section of MeS spectrum always located in the ReS. As illustrated in Figure 3, the local spectrum is defined as a part of the original ReS, which will remain in the latter MeS (after loading strain) and have high similarity to a segment of the MeS. So the improvement of the similarity can be achieved through the utilization of the local spectrum of high similarity. The appearance of multiple and fake peaks can be effectively avoided under a large strain load.

**Figure 5:**
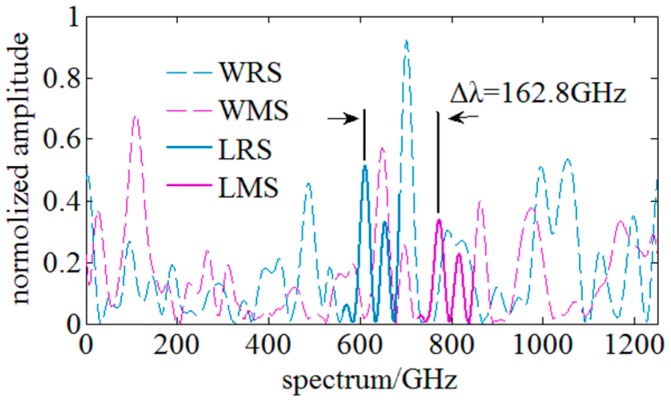
Schematic of local spectrum similarity characteristics. WRS: whole reference spectrum; WMS: whole measurement spectrum; LRS: local reference spectrum; LMS: matched local measurement spectrum; ∆λ: the corresponding spectrum shift.

Figure 5 illustrates the MeS and ReS of a distributed sensing gauge in 3 mm length under a strain of 1000 με and it indicates that a segment of the MeS (marked in solid purple line) has high similarity with the shifted ReS segment (marked in solid blue line), even though it is hard to identify the similarity and spectrum shift between the whole MeS (marked in dotted purple line) and ReS (marked in dotted purple line). Therefore, the high local spectrum similarity characteristics are experimentally verified.

**Figure 6:**
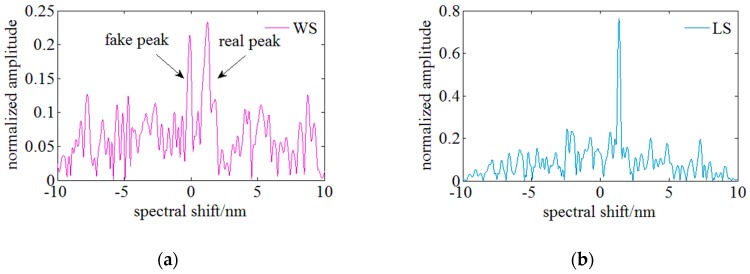
Comparison between cross-correlation results using different algorithms. (**a**) Whole spectrum (WS) algorithm. (**b**) Local spectrum (LS) algorithm.

During this experiment, a segment of ReS is selected as the local ReS (LRS) and the corresponding local MeS (LMS) with the highest similarity is found by comparing the similarity among the LRS and each LMS. The Pearson correlation coefficient is utilized to evaluate the similarity. Then, the corresponding spectrum shift can be achieved by the wavelength difference between the LRS and the matched LMS. Figure 6 shows the cross-correlation results of the whole spectra and local spectra. The comparison results indicate that the cross-correlation result of whole spectra has fake peaks and the similarity is lower than 0.25. However, a single peak of a high SNR is achieved through the cross-correlation of the local spectra and the similarity is ~0.8 which is raised by 4-fold. It is experimentally verified that utilization of the local spectrum similarity characteristics can effectively suppress the appearance of multi and fake peaks, and improve the similarity and the SNR of the cross-correlation results, so this method can achieve a higher strain measurable range using a narrow swept spectrum range.

### 2.2. The Similarity Evaluation Function Based on the Least-Square Method

Based on the local similarity characteristics of the RS spectrum, the spectrum shift between the LRS and LMS can be achieved through matching the LRS of the highest similarity in the MeS. Therefore, similarity evaluating function determines the accuracy of the achieved spectrum shift and data processing rate. In conventional OFDR methods, the evaluation criterion of similarity is on the basis of the Pearson correlation coefficient. The Pearson correlation coefficient cannot fully express the trend between the LRS and LMS because it is only a measure of the linear correlation [8]. Besides, the Pearson correlation coefficient cannot maintain a high performance when the distributed strain is over 2000 με, even if the similarity is improved by local spectrum characteristics. Multiple and fake peaks will appear in the similarity evaluation results and affect matching the LRS on the MeS, so the similarity evaluating function must be researched and improved. The cross-correlation is invalid in measuring a larger strain, so similarity between reference and measurement spectra is used to find their shift. In the conventional method, the Pearson correlation coefficient can reflect the correlation or similarity between two signals. The sequence shift represents spectrum shift. The Pearson correlation coefficient is written as:

(4) $$r(y_{{\mathrm{LRS}}_{i}},y_{{\mathrm{LMS}}_{j}})=\frac{\mathrm{cov}(y_{{\mathrm{LRS}}_{i}},y_{{\mathrm{LMS}}_{j}})}{\sqrt{\mathrm{var}\left[y_{{\mathrm{LRS}}_{i}}\right]\mathrm{var}\left[y_{{\mathrm{LMS}}_{j}}\right]}}$$

where, $\mathrm{cov}(y_{{\mathrm{LRS}}_{i}},y_{{\mathrm{LMS}}_{j}})$ is the covariance of spectra sequences $y_{{\mathrm{LRS}}_{i}}$ and $y_{{\mathrm{LMS}}_{j}}$; $\mathrm{var}\left[y_{{\mathrm{LRS}}_{i}}\right]$ and $\mathrm{var}\left[y_{{\mathrm{LMS}}_{j}}\right]$ are the variance of spectra sequences $y_{{\mathrm{LRS}}_{i}}$ and $y_{{\mathrm{LMS}}_{j}}$.

The least squares function is usually utilized to evaluate the approximating degree of two curves and achieve the fitting function [9,10]. On the other hand, the least squares function can also represent the Euclidean distance between two curves. In the match of the LRS in the LMS, the trend of these two spectra is more considerable and the least squares function is therefore more suitable for evaluating the spectrum similarity. It can be concluded that when the residual sum of squares reaches the lowest, the trend bias between spectra is smallest. A similarity evaluation function based on the least-square method is thus proposed. Furthermore, there are two factors need to be taken into consideration for the practical application: (1) the similarity between each LMS and a fixed LRS is needed to be evaluated and compared; (2) two local spectrum segments with a large amplitude difference may influence similarity evaluation badly. Therefore, normalization operation is needed before each evaluation function. The similarity evaluation function is constructed by residual sum of squares between normalized LMS and LRS:

(5) $$v(j)=\sum_{i=1}^{n-j}{\left[y_{\mathrm{LRS}}(i)-y_{\mathrm{LMS}}(i+j)\right]}^{2}$$

wherein, $y_{\mathrm{LRS}}(i)$ and $y_{\mathrm{LMS}}(i)$ are the local spectrum sequences and the sequence length is *n* (note: the data is discrete and variables of the above function are also discrete).

The sum of the squares of the residuals v between the fixed LRS and each LMS can be considered as a similarity index in the matching procedure. The value of v reaches its minimum when the most similar spectrum is matched.

**Figure 7:**
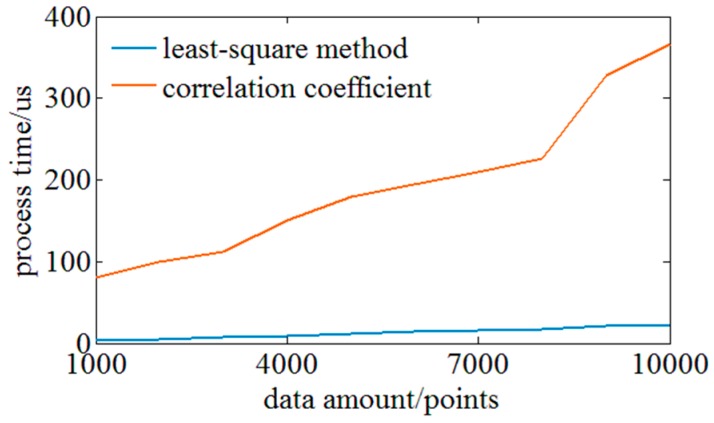
Comparison of time consumption between evaluation function based on least-squares method and evaluation function based on Pearson correlation coefficients.

Next, the performances of the similarity evaluation function based on the Pearson correlation coefficient and the least squares function are compared experimentally. Experiments on processing rate are conducted firstly. The time consumption of the two evaluation functions in MATLAB is compared when processing the data amount ranges from 1000 to 10,000. Figure 7 shows the comparison results and it indicates that the processing rate of the similarity evaluation function based on the least squares is at least 10 times faster. Furthermore, the evaluation method based on the least squares takes more advantage in terms of the growth rate of time consumption to data amount, which has benefits on processing the distributed sensing of a high density and resolution.

**Figure 8:**
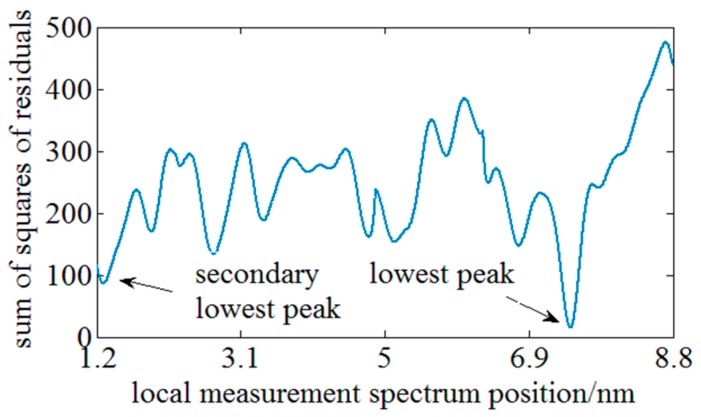
The diagram of the residual sum of squares between the fixed local ReS and each local MeS.

Subsequently, the performance of the two evaluation functions on improving the similarity is compared experimentally. SNR is taken as the index for comparing the similarity improvement achieved by the different methods. The definition of SNR for the conventional evaluation function is the amplitude ratio of the highest peak to the secondary peak in the cross-correlation results wherein the highest peak represents the available spectrum shift signal and the secondary peak represents the noise signal. According to this definition, SNR of the whole and local spectrum cross-correlation result in Figure 6 is respectively ~1.1 and ~3.1. By employing the local spectrum similarity characteristics, the SNR is raised by 3-fold. Considering the true peak value is the minimum sum of squares of residuals, the definition of SNR of the latter method is the ratio of the secondary lowest peak amplitude to the lowest peak amplitude. Figure 8 illustrates the result of the method based on the least squares function and the SNR is over than 7. The similarity is further improved by 2-fold through combining the local spectrum similarity characteristics with the evaluation method based on the least squares function, so the improvement of the proposed method on the similarity can be experimentally verified.

### 2.3. The Proposed OFDR Method

**Figure 9:**
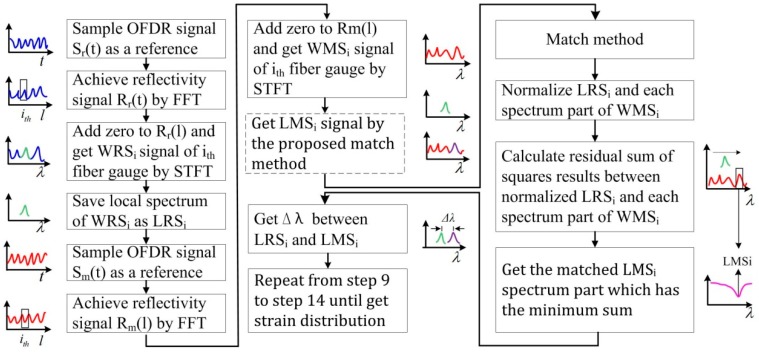
The flow diagram of OFDR strain discrimination processing algorithm based on high similarity local spectra. OFDR: optical frequency-domain reflectometry; Sr(*t*): the reference OFDR signal; Rr(*l*) the reference OFDR reflectivity signal; WRSi: whole reference spectrum of i-th fiber gauge; LRSi: a segment of WRSi; Sm(*t*): the measurement OFDR signal; Rm(*l*) the meaurement OFDR reflectivity signal; WMSi: whole measurement spectrum of *i*-th fiber gauge; LMSi: a segment of WMSi which has the minimum residual sum of squares.

The combination between the local spectrum similarity characteristics with the evaluation method based on the least squares function would enhance the similarity under a large distributed strain and the processing rate. An OFDR processing algorithm is thus proposed for achieving a high speed and a large measurable range of the distributed strain sensing. The algorithm procedures are illustrated in Figure 9 and the processing is as follows (note: the data is discrete and variables of the following function are also discrete):

(1) Sample OFDR signal $S_{r}(t)$ in a SMF in unstressed status or under a known distributed strain and save it as the reference OFDR signal.
(2) Transform the reference OFDR signal $S_{r}(t)$ into distance domain and achieve the reflectivity signal $R_{r}(l)$ along the SMF via FFT.
(3) Extract the *i*-th signal data $R_{ri}(l)$ located at a certain position wherein data length determines the spatial resolution of the fiber gauge.
(4) Add zeros to $R_{ri}(l)$ to achieve $Z_{ri}(l)$ for offsetting the loss of spectral resolution which is caused by the data extraction. The zero number is determined by selected spectrum or strain resolution. The spectrum resolution is calculated by dividing experimental spectrum range by the length of $Z_{ri}(l)$.
(5) Transform $Z_{ri}(l)$ into spectrum domain and achieve the WRSi of the *i*-th fiber gauge via STFT.
(6) Save a segment located at a certain position of each WRSi as LRSi and the length of the segment is *N*.
(7) Sample OFDR signal $S_{m}(t)$ under with unknown distributed strain as the measurement OFDR signal.
(8) Transform measurement OFDR signal *S_m_*(*t*) into the distance domain and achieve the reflectivity signal *R_m_*(*l*) along the SMF via FFT.
(9) Extract the *i*-th signal data $R_{mi}(l)$ located at a certain position wherein data length determines the spatial resolution of the fiber gauge.
(10) Add zeros to $R_{mi}(l)$ to achieve $Z_{mi}(l)$.
(11) Transform $Z_{mi}(l)$ into the spectrum domain and achieve the WMSi of the *i*-th fiber gauge via STFT.
(12) Match the LRSi with the most similar spectrum segment of the WMSi by searching the minimum residual sum of squares:
  (6) $$\begin{array}{l} L(n)=\sum_{m=1}^{N}{\left[y_{\mathrm{LRS}}(m)-y_{\mathrm{WMS}}(m+n)\right]}^{2},\;n=0,1\cdots M-N\\ \min\;L(n_{m}) \end{array}$$
  where, $y_{\mathrm{LRS}}(m)$ and $y_{\mathrm{WMS}}(m)$ are the local reference spectrum and measurement spectrum, *N* is the length of local reference spectrum and *M* is the length of measurement spectrum.
(13) Calculate the spectrum shift Δλ by comparing the wavelength shift between the matched LMSi and LRSi:
  (7) $$\Delta \lambda =n_{m}-\frac{M}{2}+\Delta n$$
  where, $n_{m}$ is the index of the best match local spectrum, *M* is the length of measurement spectrum and Δn is the internal shift between the local reference and measurement spectra.
(14) Calculate the corresponding Δε using the linear relationship between the spectrum shift and distributed strain.
(15) Repeat the process form (9) to (14) and achieve the full distributed strain along the SMF.

In the proposed method, both positive and negative strain can be demodulated. The local reference spectrum segment is matched from the start to the end of the measurement spectrum. So the positive and negative shift of the matched local measurement spectrum segment respecting to the local reference spectrum can be achieved.

## 3. Experiments and Discussion

In this section, experimental configuration and experiments are demonstrated. First, the OFDR distributed sensing system is introduced in detailed. Then, investigations on the influences of local spectrum setup are performed for optimizing the match. Finally, performance tests of the proposed OFDR methods are performed.

### 3.1. Experimental Setups

**Figure 10:**
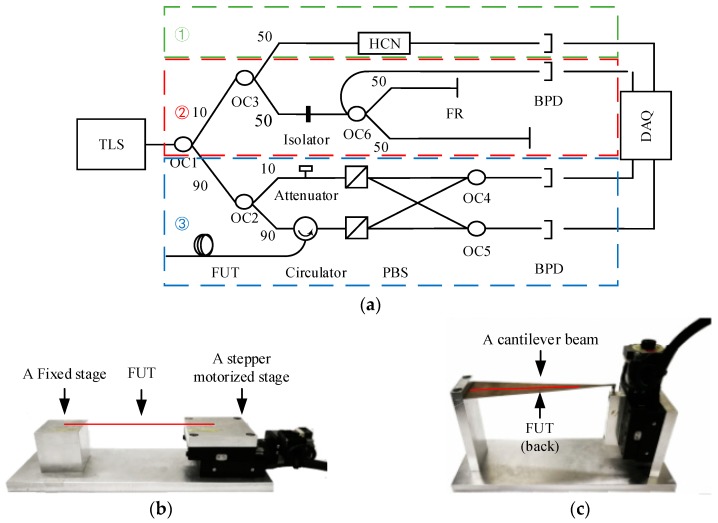
Schematic of OFDR distributed sensing system and experimental setups. (**a**) Configuration of OFDR distributed sensing system. (**b**) Experimental setups of stretching fiber device for generating large distributed strain. (**c**) Experimental setups of cantilever device for generating micro distributed strain. TLS: tunable laser source; OC: optical coupler; FR: Faraday reflector; PBS: polarization beam splitter; BPD: balanced photodetector; FUT: fiber under test; DAQ: data acquisition.

Figure 10a illustrates the experimental setups of the OFDR distributed sensing system [11]. The data acquisition (DAQ) card is PCI-1714 produced by Advantech (Taibei, Taiwan). The balanced photodetector (BPD) is a polarization dependent balanced detector INT-POL-1550 produced by Thorlabs (Newton, NJ, USA). The external cavity tunable laser source (TLS) is a T100R produced by Yenista (Quebec, QC, Canada). The HCN gas cell is H13C14N produced by Wavelength Reference (Corvallis, OR, USA) which is equivalent of the NIST SRM 2519. The other optical fiber devices are all produced by Advance Fiber Resources (Zhuhai, Guangdong, China).

Figure 10b,c show the devices for generating controllable distributed strain. A stretching fiber device and a cantilever device are respectively for testing large and small distributed strain. A stepper motorized stage KXC06 by Suruga Seiki (Shizuoka, Shizuoka, Japan) is employed to generate micro displacements downwards to 50 nm. In Figure 10b, the fiber under test (FUT) is a section of sensing fiber between 1.3 m to 1.6 m and it is glued on the stepper motorized stage and a fixed stage. A maximum distributed strain up to 3000 με can be achieved. Besides, the computing time based on Pearson coefficient and proposed method processing 200 distributed sensing gauges is ~240 ms and ~25 ms, respectively. The sweep time for conventional method with a sweep range of 30 nm is 750 ms. By this method, a sweep range of 10 nm would save 500 ms based on the configuration of the personal computer used to run the program which is as follows: the processor frequency is 3.2 GHz and core number is four. RAM is 16 GHz.

**Figure 11:**
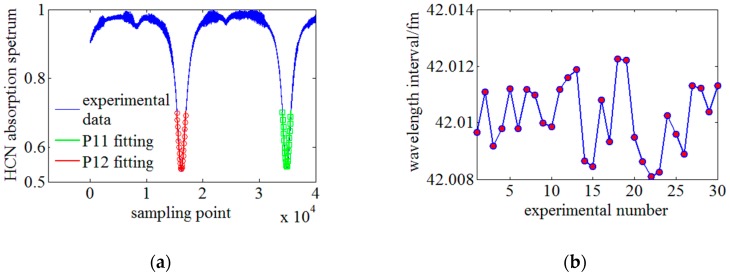
The experimental on the wavelength calibration configuration. (**a**) The absorption spectrum between P11 and P12 peak of the HCN gas cell and the Vogt fitting results. (**b**) Experimental results of equal-wavelength-sampling interval. P11: the 11-th absorption peak of the HCN; P12: the 12th absorption peak of the HCN.

In Figure 10c, the fiber under test (FUT) is a section of sensing fiber between 0.85 m to 0.9 m and it is glued on a silico-manganese bronze cantilever. The accuracy of the generated micro strain is higher than 10 με. The wavelength repeatability of TLS is ~25 pm and is much worse than the requirement of strain resolution. On the other hand, spatial resolution is related to the nonlinearity of frequency sweeping and equal-wavelength-sampling or equal-spectrum-sampling are thus highly required. The equal-wavelength-sampling interval is determined by the length difference of the auxiliary interference arms which cannot be precisely measured, so a wavelength calibration configuration consisting of the auxiliary interferometer and HCN gas cell is designed to improve the absolute wavelength accuracy to sub pm and calibration the wavelength-sampling interval [12]. During the experiments, the tunable range of the TLS is from 1540 to 1550 nm and the sweep velocity is ~40 nm/s. The normalized absorption curve of the HCN gas cell between 1549 nm and 1551 nm are illustrated in Figure 11a. The absorption central indexes (P11, 1549.7302 nm; P12, 1550.5149 nm) are achieved through fitting the curve into a Vogt function and the equal-wavelength-sampling interval can be derived as:

(8) $$\Delta \lambda =\frac{\lambda_{2}-\lambda_{1}}{N_{2}-N_{1}}=\frac{\Delta \lambda_{2-1}}{\Delta N_{2-1}}$$

where, $\lambda_{1}$ is the $i_{\mathrm{th}}$ absolute absorption peak wavelength of the HCN gas cell, $N_{i}$ is the index of the $i_{\mathrm{th}}$ peak and Δλ is the derived wavelength interval.

The length difference of the auxiliary interference arms is affected by slow temperature drifts. The wavelength calibration is performed twice every minute. Figure 11b shows the calibration results of 30 times. Based on the experimental results, the equal-wavelength-sampling interval is 42.012 fm and the calibration repeatability is 0.041 fm. It can be inferred that the wavelength calibration configuration is stable and robust.

### 3.2. Optimization of the Proposed OFDR Distributed Sensing Method

Although it has been verified that using the local spectrum can significantly improve SNR and similarity, it is necessary to investigate the influence of the local spectrum length and position on the performance of the distributed strain sensing. The length of the local spectrum refers to the wavelength range of the local spectrum. The position of the local spectrum refers to the local spectrum center position relative to the whole spectrum.

**Figure 12:**
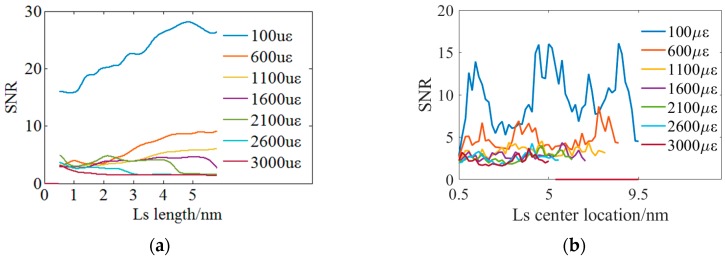
Experiments on the optimizing the local spectrum (LS) parameters. (**a**) Experimental results of optimizing the local spectrum length. (**b**) Experimental results of optimizing the local spectrum center.

The experiments are conducted 30 times under a same conditions and the SNR is an averaged result of the distributed sensing region of 0.2 m subjected to a strain. The statistical results are shown in Figure 12. Figure 12a shows the influences of the local spectrum length on the SNR when the local spectrum position is set at the middle of the whole spectrum. SNR is defined as the amplitude ratio of the secondary lowest peak amplitude to the lowest peak amplitude when the local spectra are all correctly matched. However, SNR is defined as zero once there is a mistaken match. Figure 12a shows that SNR is zero when the local spectrum length is less than ~0.5 nm because the spectrum characteristics are not sufficient. SNR increases with the local spectrum length under a distributed strain of less than 1600 με as a result of a higher match. It suggests that the longer local spectrum contains more characteristics to improve the accuracy of spectra matching and the influence of new spectrum segment is relatively weak. However, SNR begins to decrease with the local spectrum length when the loaded strain exceeds 2100 με. Besides, as illustrated in Figure 4, a red or blue spectrum shift of ~3.8 nm induced by tensile or compressive distributed strain of 3000 με and the length of the LRS and LMS spectrum segments keeping a high similarity is ~1.0 nm. Finally, the length of the local spectrum is set as 1.0 nm.

Then, the influence of the local spectrum position is experimentally investigated and the local spectrum length is 1.0 nm. Figure 12b illustrates the experimental results and it indicates that SNR is hardly influenced by the spectrum position. However, the LMS desired to be matched are easily shifted out of the WMS as a result of the different spectrum shift direction of the tensile and compressive distributed strain. Considering the measurable range of the tensile or compressive distributed strain, the local spectrum position is finally set as 5.0 nm at the middle of the whole spectrum. SNR is most stable at the center of the swept. In our method, a spectrum segment is chosen in the reference spectrum as the local reference spectrum (LRS). Then, the measurement spectrum may be considered as a shift reference spectrum. This method matches the LRS segment on the measurement spectrum and finds the shift between the center of LRS and the matched spectrum segment in the measurement spectrum. No matter whether under stretching or compression, the center spectrum segment in the reference spectrum is the last to lose its similar segment part in the measurement spectrum, so the SNR would not drop in the center.

Based on the results of Figure 12, the spectrum length and location should be carefully selected. Excessive reduction of cross-correlation points would yield a lower SNR, however, appropriate reduction points could improve the SNR. So a conclusion about how to enhance the similarity between reference and measurement spectra and improve the SNR during the determination of spectrum shifts. This is a great contribution and significant discovery of this paper.

### 3.3. Experiments on Testing the Distributed Sensing Performances of the Proposed Method

The performances of the proposed OFDR method are experimentally tested and compared with conventional OFDR methods, including the improvement of SNR, the sensing performance of large distributed strain, resolution of the distributed strain and the repeatability across the full strain range.

**Figure 13:**
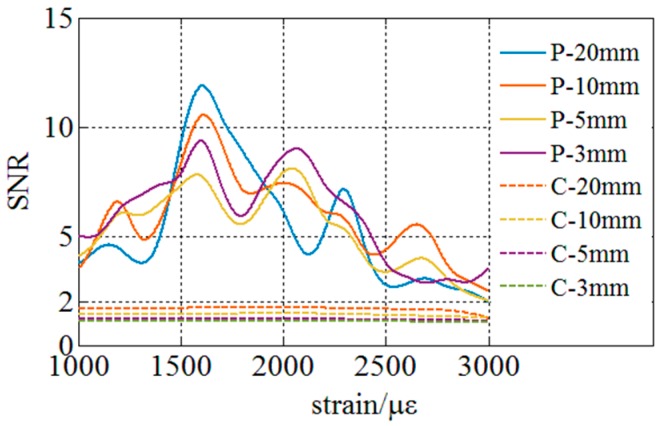
Experiment on comparing SNR of the similarity evaluating function between the proposed (P) and the conventional method (C) under different spatial resolutions.

Experiments on the improvement of SNR are conducted first. This experiment is aimed at comparing the SNR of the similarity evaluating function between the proposed and the conventional method during the determination of spectrum shift. In the conventional method based on cross-correlation, SNR is defined as the amplitude ratio of the secondary highest peak amplitude to the highest peak amplitude when the local spectra are all correctly matched. SNR is defined as zero once fake peak or multi-peaks exits in cross-correlation and wavelength shift cannot be distinguished the cross-correlation wavelength shift can’t be determined. Experimental results shown in Figure 13 are derived from the distributed sensing fiber in a range from 1.3 m to 1.6 m. The experiments are conducted 30 times under a same condition and the SNR is an averaged result of the distributed sensing region of 0.2 m subjected to a strain. The statistical results are shown in Figure 13. SNR is defined as zero once there is a mistaken match. The dotted lines represent the SNR under different spatial resolutions using the conventional method dealing with cross-correlation, and the solid lines represent the SNR under different spatial resolutions using the proposed method dealing with the least-square method. SNR achieved by the conventional method is lower than 2 when the distributed strain is larger than 1000 με and multi or fake peaks begin to appear which leads to the failure of the distributed strain sensing. However, SNR of the proposed method is in a range from 2 to 12 which verifies that it is robust for sensing large distributed strain. In summary, the proposed method can improve SNR of the similarity evaluating function under small distributed strain and make the distributed strain sensing of larger than 1000 με range possible when the conventional method is thoroughly invalid. The proposed method could raise the strain measurable range to 3000 με which exceeds the reported OFDR methods [6].

**Figure 14:**
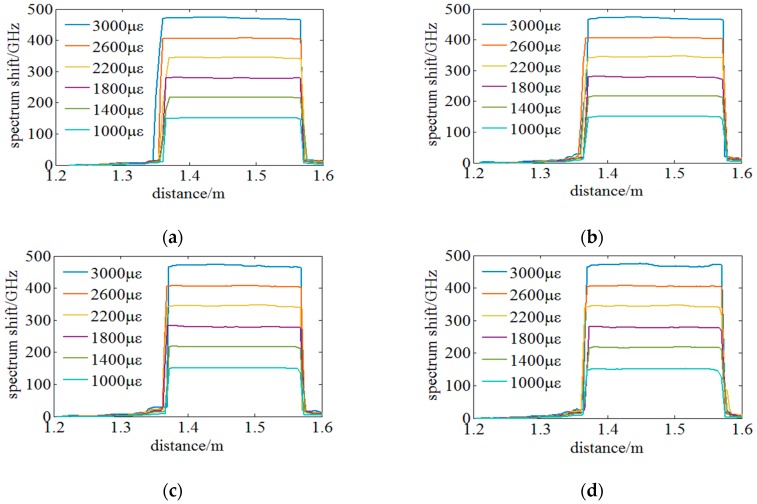
Experiments on sensing large distributed strain under different spatial resolutions: (**a**) 20 mm, (**b**) 10 mm, (**c**) 5 mm and (**d**) and 3 mm.

Then, experiments on the sensing performance of a large distributed strain under different spatial resolutions are conducted. Distributed strains from 1000 µε to 3000 µε in an interval of 400 µε are loaded on the FUT between 1.3 m to 1.6 m. The proposed method is utilized to process sensing signal and senses the distributed strain. The experimental results are illustrated in Figure 14. It indicates that the strain measurable range of the proposed method can reach 3000 µε under a 3 mm high spatial resolution and there is no mistaken point in the distributed sensing region. On the other hand, the smoothness of the distributed strain gets worse when the distributed strain becomes larger and the spatial resolution becomes higher. It would be caused by that SNR is influence by the amount of distributed sensing data and similarity. Based on the theory of STFT, the resolution of spatial and spectrum has an interaction relationship. The similarity degenerates due to new spectrum segments appearing in the whole MeS. Furthermore, the rising or falling phase of the distributed strain under a spatial resolution of 20 mm is longer than that under a higher spatial resolution. It is mainly induced by the STFT window containing more or less data points reflecting the distributed strain when the STFT windows are sliding in and out of the strain region. It can be concluded that fake peaks and multiple peaks are thoroughly eliminated by the proposed OFDR method and the strain measurable range up to 3000 µε is achieved under a highest spatial resolution downwards to 3 mm.

According to Figure 14, the spectrum shift varies linearly with the increase of the distributed strain and so sensitivity can be calibrated as follows. The average spectrum shift of the fiber gauges within the sensing region is extracted under different strains and is fitted into a linear function. The sensing curve can be written as:

(9) Δλ=kε

where, Δλ is the spectrum shift, k is the sensitivity coefficient and ε is the strain.

**Figure 15:**
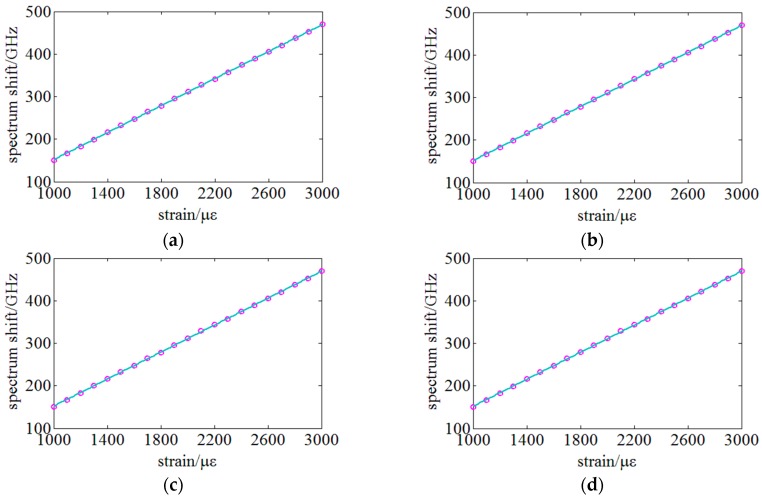
Experiments on the sensing sensitivity of the distributed strain under different spatial resolutions: (**a**) 20 mm, (**b**) 10 mm, (**c**) 5 mm and (**d**) and 3 mm.

Figure 15 illustrates the results of sensing sensitivity under different spatial resolutions of 20, 10, 5 and 3 mm are respectively 0.1586, 0.1587, 0.1586 and 0.1587 GHz/µε. The diversity of sensitivities under different spatial resolutions is less than 0.03%. The sensing curves or the relationship between spectrum shifts and strain are quite stable under the same condition even with different spatial resolutions. Furthermore, the nonlinearity of the sensing curves is less than 0.5%.

**Figure 16:**
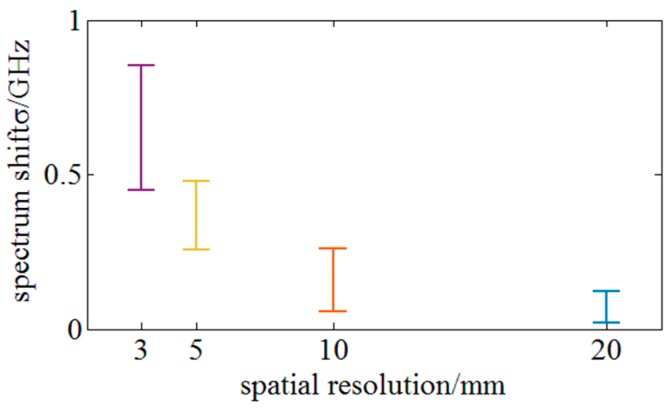
Experiments on the repeatability across the full strain range under different spatial resolutions.

The repeatability across the full strain range can be derived from the Figure 14. Repeatability across strain the full range refers to average repeatability over full strain range 3000 µε. Repeatability is measured and reflects 2σ standard deviation from the stable distributed sensing region. Figure 16 indicates that the repeatability across the full strain range under different spatial resolutions of 3, 5, 10 and 20 mm is respectively 0.85, 0.48, 0.26 and 0.12 GHz. The result of a 5 mm gauge length is better than that of LUNA ODiSI-B (0.79 GHz) representing the leading level of the OFDR.

**Figure 17:**
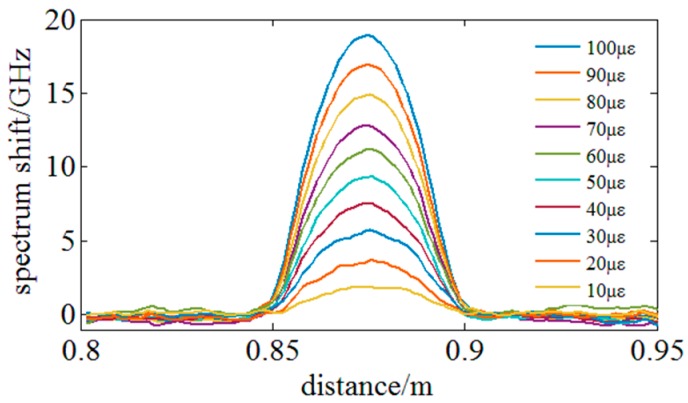
Experiments on sensing micro distributed strain or strain resolution under the highest spatial resolution of 3 mm.

Finally, the strain resolution of the proposed method is experimentally tested. In Figure 10c, the sensing fiber between 0.85 m to 0.9 m is glued on a silico-manganese bronze cantilever and a stepping motor is utilized to generate micro strain steps. Figure 17 illustrates the experimental results and it indicates that micro strain steps of 10 με (limited by the device generating strain) can be clearly distinguished under a high spatial resolution of 3 mm.

On the other hand, the noise floor in the region of sensing distributed strain is about ±2 με which means that the strain resolution is on the level of 2 με. Furthermore, the noise floor out of the region of sensing distributed strain is lower than ±0.4 GHz which could represent the repeatability at zero strain and is better than ±0.75 GHz achieved by the LUNA ODiSI-B.

## 4. Conclusions

In this paper, the restrictions on the measurable range strain using a narrow swept spectrum range are investigated and a new similarity evaluation function is built to improve the data processing rate and SNR during determination of the spectrum shift. The new spectrum segment in the MeS spectrum without similarity to ReS segment is the primary reason causing the similarity degeneration between ReS and MeS. The local spectrum characteristics are found and SNR of the similarity evaluating results is enhanced by the proposed method so that the fake peaks and multi peaks are thoroughly eliminated. Then, a new similarity evaluation function based on least-square method is built to replace the Pearson correlation coefficient and the amount of calculation is decreased significantly. By combining these two variations, the proposed OFDR method matches the local ReS in the MeS to find the most similar MeS segment wherein the least-squares method is employed to evaluate similarity. By the proposed method, the measurable strain range can reach 3000 µε under a highest spatial resolution of 3 mm when the sweep wavelength range is only 10 nm. The data processing rate is raised by at least 10 times than that employing Pearson correlation coefficients. Experimental results indicate that a nonlinearity of less than 0.5%, a strain resolution of better than 10 µε, a repeatability at zero strain of below ±0.4 GHz and a repeatability across the full strain range of less than 0.85 GHz are achievable under a highest spatial resolution of 3 mm. This work solves the problem of the invalidation of conventional methods when measuring a large strain and the failure to find the true cross-correlation peak when the sweep is insufficient. The performances of the proposed method are better than those of a LUNA ODiSI-B device representing the leading level of OFDR.
